# Metal Ion–Engineered Carbon Quantum Dots From Hazelnut Shell via Solid‐State Synthesis for Efficient OLED Devices

**DOI:** 10.1002/bio.70503

**Published:** 2026-05-12

**Authors:** Fatmanur Uyumaz Cengiz, Figen Türksoy, Emine Tekin, Güldem Utkan, Erhan Şükrü Cengiz, Görkem Yumuşak, Memet Vezir Kahraman

**Affiliations:** ^1^ Department of Chemistry, Faculty of Science Marmara University Istanbul Türkiye; ^2^ Materials and Process Technologies TÜBİTAK Marmara Research Center Kocaeli Türkiye; ^3^ Department of Chemistry, Faculty of Science and Arts Duzce University Düzce Türkiye; ^4^ SUNUM Nanotechnology Research Center Sabanci University Istanbul Türkiye; ^5^ Department of Metallurgical and Materials Engineering Marmara University Istanbul Türkiye

**Keywords:** carbon quantum dots, hazelnut shell biomass, OLED, solid‐state synthesis, sustainable materials

## Abstract

In this study, we report a scalable and green solid‐state synthesis of nitrogen‐doped carbon quantum dots (CQDs) from hazelnut shell biomass, using citric acid and urea as carbon and nitrogen sources. Controlled BaCl_2_ and ZnCl_2_ doping was applied to tailor nucleation, crystallinity, and surface chemistry. Structural analyses (FTIR, XRD, STEM, XPS, and DLS) revealed that BaCl_2_‐assisted CQDs exhibited higher graphitization, narrower size distribution (7–13 nm), and fewer defects, while ZnCl_2_‐assisted CQDs showed more amorphous and heteroatom‐rich surfaces. Optical measurements indicated strong π–π* absorption (≈280 nm), bright blue emission (*λ*_em_ 405–412 nm), and quantum yields of 63.4% (BaCl_2_) and 50.1% (ZnCl_2_), with > 95% stability after 30 days. When used as OLED emissive layers, BaCl_2_‐CQDs achieved a luminous efficiency of 0.75 cd A^−1^, nearly four times that of ZnCl_2_‐CQDs (0.20 cd A^−1^), despite lower maximum luminance (48.9 vs. 308.1 cd m^−2^). These results highlight metal ion–assisted nucleation as an effective strategy to engineer CQD properties and enhance device performance, paving the way for sustainable, scalable OLED technologies.

## Graphical abstract

Solid‐state synthesis of nitrogen‐doped carbon quantum dots (CQDs) from hazelnut shell enables tunable structure and enhanced photoluminescence via metal‐ion engineering. Ba^2+^‐assisted CQDs exhibit improved graphitization and superior OLED efficiency, demonstrating a sustainable route for high‐performance optoelectronic materials.

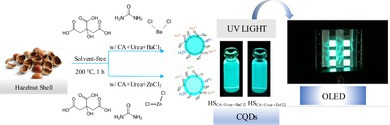

## 1 Introduction

Organic light‐emitting diodes (OLEDs) are an advanced class of optoelectronic devices in which light is produced through the electroluminescence of organic semiconductors. Under an applied electric field, electrons and holes recombine radiatively within the emissive layer, resulting in photon emission. OLEDs can be fabricated either from small molecules or conjugated polymers, the latter enabling solution processing and compatibility with scalable printing methods. Despite this advantage, small‐molecule OLEDs still dominate commercial production due to their superior efficiency and device longevity [1, 2].

The self‐emissive nature of OLED pixels provides true blacks, high‐contrast ratios, and vivid color reproduction while eliminating the need for a backlight, which reduces power consumption and allows ultrathin, lightweight device architectures [3, 4]. These features have led to their rapid adoption in smartphones, televisions, and emerging flexible and foldable display technologies. However, challenges such as the limited operational stability of blue emitters, sensitivity to oxygen and moisture, and high manufacturing costs continue to limit broader commercialization. As a result, there is growing interest in exploring novel, sustainable emissive materials that combine high efficiency with stability under ambient conditions [5, 6, 7].

Within this search for next‐generation emitters, carbon quantum dots (CQDs) have gained significant attention as promising zero‐dimensional carbon nanomaterials with unique photophysical properties. Typically smaller than 10 nm, CQDs exhibit tunable photoluminescence (PL) governed by quantum confinement and surface states [8, 9, 10]. They can be synthesized through versatile bottom‐up approaches, including hydrothermal, solvothermal, and microwave‐assisted methods, using diverse carbon sources such as small organic molecules or renewable biomass [11, 12]. Their water dispersibility, chemical stability, and low toxicity make them environmentally friendly alternatives to heavy‐metal‐based quantum dots, suitable for bioimaging, sensing, catalysis, and optoelectronics [13, 14].

Recent studies have demonstrated the potential of CQDs in OLED architectures, where their bright and tunable emission, surface functionalizability, and solution processability allow their use as charge‐transport layers and even as emissive components. By adjusting particle size and heteroatom doping, CQDs can emit across the visible spectrum, enabling full‐color OLED fabrication. Moreover, their thermally activated delayed fluorescence characteristics improve exciton harvesting and enhance external quantum efficiency (EQE), opening a route to cost‐effective and sustainable OLED technologies [15, 16, 17, 18].

Building on this potential, attention has increasingly turned toward sustainable and low‐cost carbon sources for CQD production. Hazelnut shells, a major agricultural by‐product particularly abundant in Türkiye, represent an attractive precursor due to their high cellulose, hemicellulose, and lignin content, which provide a carbon‐rich framework facilitating functional group formation during carbonization. This results in CQDs with excellent PL and quantum yield [19, 20, 21, 22]. Furthermore, hazelnut shells can be processed through simple, scalable, and cost‐effective methods such as hydrothermal carbonization or pyrolysis, with tunable synthesis parameters to precisely control emission properties, critical for achieving the color purity and brightness demanded in advanced display technologies [23, 24].

In the present study, we report a facile solid‐state strategy to produce nitrogen‐doped CQDs (N‐CQDs) from hazelnut shell powder, valorizing an abundant local biomass waste stream. Citric acid was used as a cocarbon precursor to enhance carbonization, while urea introduced nitrogen functionalities and improved surface passivation. Additionally, controlled BaCl_2_ and ZnCl_2_ doping was employed to modulate crystallinity and tune optical properties. The resulting CQDs exhibited bright, excitation‐dependent PL, high quantum yield, and excellent colloidal stability. When incorporated as the emissive layer in OLED prototypes, these CQDs produced robust luminescence and promising device performance. This work demonstrates the feasibility of converting Türkiye's plentiful hazelnut shell waste into high‐value emissive nanomaterials and establishes a scalable route toward environmentally responsible, circular economy–oriented OLED fabrication.

## 2 Experimental

### 2.1 Materials

Hazelnut shell was purchased from Koruma Klor, Türkiye. Citric acid, urea, BaCl_2_, and ZnCl_2_ were purchased from Sigma‐Aldrich, the Netherlands.

### 2.2 Synthesis of CQDs

**FIGURE 1:**
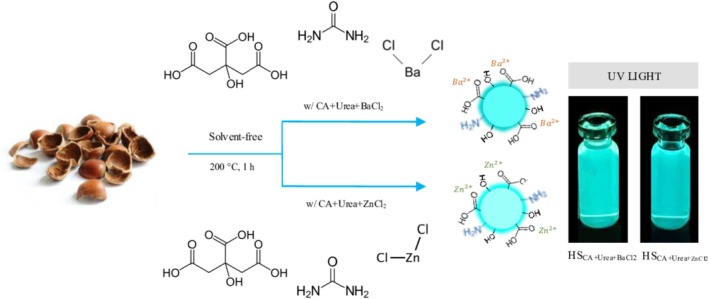
Schematic representation of the solid‐state synthesis process of N‐CQDs derived from hazelnut shell.

To obtain N‐CQDs, we adopted a direct thermochemical conversion strategy that relies on the inherent carbon richness of hazelnut shells. Initially, pulverized hazelnut shell biomass (0.75 g) was combined with citric acid (0.25 g) as an auxiliary carbon precursor, urea (1.0 g) as the nitrogen dopant and passivating agent, and either BaCl_2_ or ZnCl_2_ (1.0 g) as structural modifiers. The mixture was carefully homogenized by manual grinding until a uniformly blended precursor was achieved. Instead of using solvents or multistep treatments, the mixture was subjected to a single‐step thermal activation at 200°C for 1 h in a convection oven. During this step, simultaneous carbonization and heteroatom incorporation took place, yielding a solid, carbon‐enriched product with a characteristic dark‐brown coloration. The obtained material was redispersed in ultrapure water (30 mL) and treated in an ultrasonic bath (40 kHz, 30 min) to promote exfoliation and achieve nanoscale dispersion. Crude suspensions were clarified by centrifugation (12,000 rpm, 20 min) to remove residual biomass and large particulates, followed by microfiltration through a 0.22‐μm membrane. To ensure removal of ionic species and low molecular weight by‐products, the filtrate was dialyzed against ultrapure water for 24 h using a 1000‐Da MWCO membrane with periodic renewal of the external water phase. The purified dispersion was then lyophilized, producing a pale, free‐flowing powder of N‐CQDs that remained stable under dry storage. For clarity, the samples obtained with BaCl_2_ and ZnCl_2_ were denoted as HS_CA+Urea+BaCl2_ and HS_CA+Urea+ZnCl2_, respectively, throughout this work. A schematic illustration summarizing this solvent‐free synthesis and purification pathway is presented in Figure 1.

## 3 Characterization

### 3.1 Characterization of CQDs

A comprehensive set of physicochemical and spectroscopic techniques was applied to elucidate the structural, optical, and surface characteristics of the hazelnut shell‐derived CQDs.

Surface chemistry was first examined by Fourier‐transform infrared (FTIR) spectroscopy using a PerkinElmer Spectrum 100 spectrometer, scanning across 4000–400 cm^−1^ to identify vibrational fingerprints associated with functional groups responsible for surface passivation. UV–visible (UV–Vis) absorption spectra were then collected on a Shimadzu UV‐2450 to probe the electronic transitions and estimate the optical bandgap of the nanodots. Steady‐state PL spectra were recorded with a Hitachi F‐7000 fluorometer at room temperature, enabling a detailed evaluation of emission profiles.

The hydrodynamic size and dispersity of the CQDs were quantified by dynamic light scattering (DLS) using a Brookhaven 90Plus analyzer. Structural ordering and crystallinity were investigated through powder X‐ray diffraction (XRD) measurements on a Philips diffractometer employing Cu Kα radiation (*λ* = 1.5406 Å). Morphological features, particle uniformity, and aggregation state were visualized via high scanning transmission electron microscopy (STEM) using a Thermo Scientific Quattro S instrument.

Elemental composition and chemical states of carbon, nitrogen, oxygen, and metal dopants were determined by X‐ray photoelectron spectroscopy (XPS) on a Thermo Fisher K‐Alpha system, providing insight into heteroatom incorporation and surface functionalization. Finally, the PL quantum yield (PL QY) was measured using a comparative method with quinine sulfate (*φ* = 0.54 in 0.1‐M H_2_SO_4_, *λ*_exc_ = 360 nm) as the standard. The quantum yield (*φ*_CQD_) was calculated from the ratio of integrated emission intensities, corrected for absorbance and refractive index according to the relation:

$$\varphi_{\mathrm{CQD}}=\varphi_{\mathrm{QS}}\times \left({Ι}_{\mathrm{CQD}}/{Ι}_{\mathrm{QS}}\right)\times (A_{\mathrm{QS}}/A_{\mathrm{CQD}}\left(\eta_{{\mathrm{CQD}}^{2}}/\eta_{{\mathrm{QS}}^{2}}\right)$$

where *I* is the integrated PL intensity, *A* is absorbance at the excitation wavelength, and *η* is the solvent refractive index. This rigorous approach enabled reliable comparison of quantum efficiency values with those reported in the literature, ensuring reproducibility and scientific validity.

### 3.2 OLED Fabrication and Characterization

Electron transport layers (ETLs), namely, tris(8‐hydroxyquinolinato)aluminum (Alq_3_, sublimed > 99.9%) and 1,3,5‐tris(1‐phenyl‐1*H*‐benzimidazol‐2‐yl)benzene (TPBi, sublimed grade, 99.995%), were procured from Sigma‐Aldrich. The hole transport material poly(3,4‐ethylenedioxythiophene)–poly(styrenesulfonate) (PEDOT:PSS) was sourced from Heraeus Clevios GmbH. Glass substrates coated with indium tin oxide (ITO) (sheet resistance: 10–15 Ω sq^−1^) were provided by LUMTEC. The configuration of the fabricated devices can be described as ITO/PEDOT:PSS/Carbon Dots (Active Layer)/ETL/LiF/Al. Carbon dot (CD) solutions were prepared in dimethylformamide (DMF) at a concentration of 20 mg mL^−1^ and deposited via spin coating to form uniform thin films. The PEDOT:PSS layer was similarly applied by spin coating and subsequently annealed to ensure optimal film quality and surface smoothness. The ETL materials, Alq_3_ (20 nm) and TPBi (20 nm), were thermally evaporated under a high vacuum of 10^−6^ mbar. In all device architectures, as cathode electrode, 1‐nm lithium fluoride (LiF) layer and a 100‐nm aluminum (Al) were sequentially deposited by thermal evaporation using a precision shadow mask under identical vacuum conditions, serving as the electron injection layer and cathode, respectively. The thicknesses of the hole transport layer (PEDOT:PSS) and the emissive CD layer were approximately 90 and 50 nm, respectively. The OLED devices were characterized using a Hamamatsu Brightness Light Distribution Measurement System (Model C9920‐11).

## 4 Results and Discussion

**FIGURE 2:**
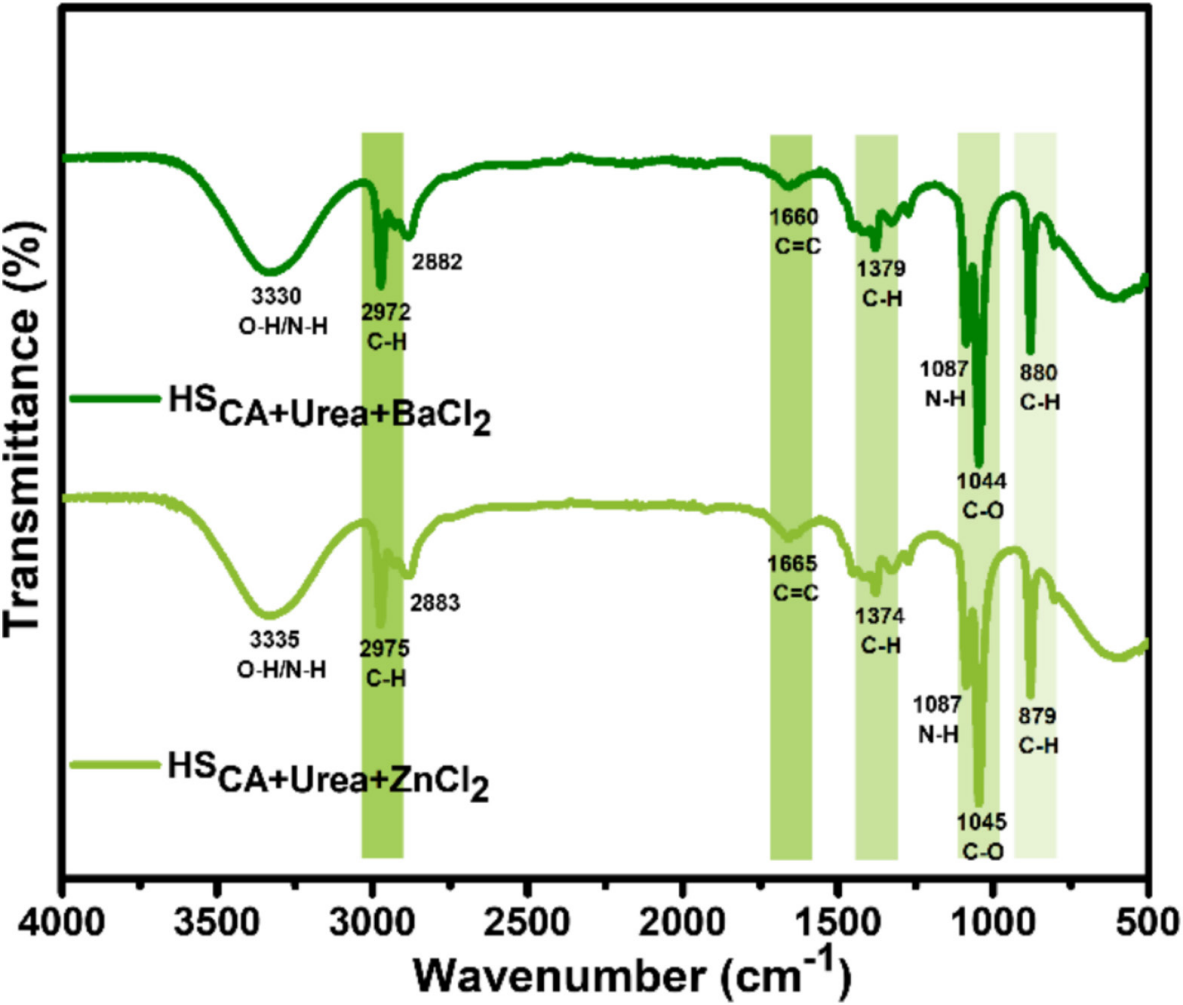
FTIR spectrum of N‐CQDs.

The FTIR spectra of HS_CA+Urea+BaCl2_ and HS_CA+Urea+ZnCl2_ systems reveal the presence of abundant oxygenated and nitrogenated functionalities, confirming successful heteroatom incorporation during the carbonization process (Figure 2). Both samples exhibit a broad band around 3330–3335 cm^−1^, corresponding to the stretching vibrations of O–H and N–H groups, indicating the retention of hydroxyl and amide functionalities from the lignocellulosic precursors and urea [25]. The characteristic C–H stretching modes appear at 2972–2975 and 2882–2883 cm^−1^, suggesting the presence of residual aliphatic moieties within the carbonaceous framework. A distinct absorption band at 1660 cm^−1^ for the BaCl_2_‐doped sample and a slightly shifted peak at 1665 cm^−1^ for the ZnCl_2_‐doped counterpart are attributed to C = C stretching vibrations, signifying the formation of conjugated sp^2^‐hybridized domains and partial aromatization during thermal treatment [26]. In both spectra, deformation vibrations at 1374–1379 cm^−1^ are assigned to C–H bending, while a strong band at 1044–1045 cm^−1^ corresponds to C–O stretching from alcohol, ether, or ester functionalities, consistent with the presence of oxygen‐rich surface groups [27]. Notably, the BaCl_2_‐doped product displays more pronounced aromatic C–H out‐of‐plane bending near 880 cm^−1^, suggesting a higher degree of graphitic ordering, whereas the ZnCl_2_‐doped sample exhibits sharper O–H/N–H and C–O bands, indicating a more amorphous carbon matrix with richer polar surface chemistry. These results corroborate the catalytic role of BaCl_2_ in enhancing carbonization and graphitization, whereas ZnCl_2_ promotes surface passivation and heteroatom retention, ultimately tuning the electronic and chemical structure of the resulting CQDs. Such tunability is crucial for optimizing the optical and electronic properties of CQDs for applications in optoelectronics and sensing.

**FIGURE 3:**
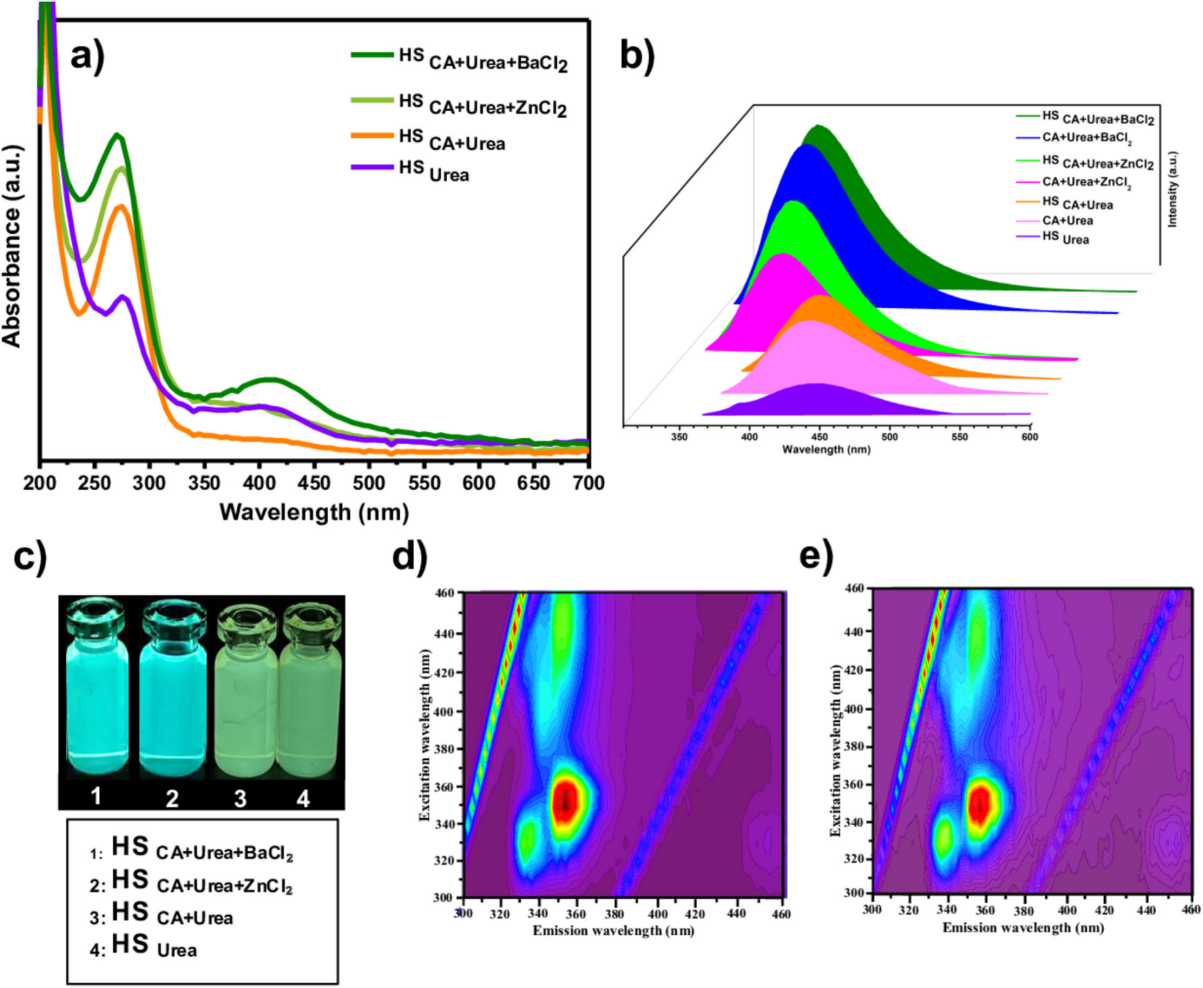
(a) UV–Vis absorption spectra (inset: photographs under UV light). (b) PL emission spectra. (c) Sample images under 365‐nm UV light. Excitation–emission matrix maps of (d) BaCl_2_‐ and (e) ZnCl_2_‐doped CQDs, confirming excitation‐dependent blue photoluminescence.

The optical properties of the CQDs synthesized from hazelnut shell biomass through four distinct precursor systems. HS_CA+Urea+BaCl2_, HS_CA+Urea+ZnCl2_, HS_CA+Urea_, and HS_Urea_ were comprehensively investigated using UV–Vis absorption and PL spectroscopy, as illustrated in Figure 3.

All samples exhibit intense absorption in the ultraviolet region, with a primary band centered at 270–280 nm, corresponding to π–π* electronic transitions of aromatic C = C bonds within sp^2^‐hybridized conjugated carbon domains [28, 29]. A less intense shoulder feature around 320–330 nm can be attributed to n–π* transitions of surface C = O and C = N moieties, indicating the presence of oxygen‐ and nitrogen‐based functional groups that act as surface emissive sites. Among the tested samples, the BaCl_2_‐assisted CQDs displayed the highest absorption intensity and the most well‐defined spectral features, implying extended π‐conjugation and enhanced graphitization. The ZnCl_2_‐assisted CQDs also exhibited strong UV absorption, albeit with a slightly broader tail toward the visible region, suggesting the coexistence of amorphous domains enriched with heteroatoms. Conversely, the HS_CA+Urea_ and HS_Urea_ samples presented significantly weaker absorption and poorly defined shoulders, reflecting a lower density of sp^2^ clusters and diminished electronic delocalization.

Under 365‐nm UV irradiation (Figure 3c), distinct differences in luminescence behavior were observed among all samples. The BaCl_2_‐assisted sample emitted an intense bright blue fluorescence, while the ZnCl_2_‐assisted sample showed a moderately strong blue emission. In contrast, the undoped systems (HS_CA+Urea_ and HS_Urea_) exhibited noticeably weaker luminescence. This contrast demonstrates that the synergistic combination of citric acid as a carbonization promoter and metal chloride as a structural modifier significantly enhances both conjugation and the formation of emissive surface states.

The PL emission spectra (Figure 3b) reveal broad, excitation‐dependent luminescence peaks centered between 400 and 420 nm upon excitation near 350 nm. The BaCl_2_‐derived CQDs exhibited the most intense and spectrally confined emission, signifying efficient radiative recombination via well‐structured sp^2^/sp^3^ interfaces and reduced nonradiative trap states [30]. The ZnCl_2_‐assisted CQDs displayed a slightly red‐shifted and broader emission profile, attributed to increased surface oxidation and more heterogeneous emissive states. The undoped CQDs, lacking either metal assistance or optimized carbonization conditions, exhibited lower emission intensities and broader features, indicating less efficient electronic transitions.

The excitation–emission matrix (EEM) maps (Figure 3d,e) further substantiate these trends. Both metal‐assisted CQDs display excitation‐dependent blue PL, with major maxima located at *λ*_ex_ = 340–360 nm and *λ*_em_ = 400–420 nm. The BaCl_2_‐assisted CQDs exhibit a compact and symmetric emission domain, whereas the ZnCl_2_‐assisted CQDs show a broader and slightly red‐shifted distribution, consistent with surface state–dominated emission mechanisms [31].

Quantitative PL QY measurements further highlight these differences. The HS_CA+Urea+BaCl2_ and HS_CA+Urea+ZnCl2_ samples exhibit QY values of 63.4% and 50.1%, respectively, using quinine sulfate in 0.1‐M H_2_SO_4_ (Φ = 0.54) as a reference. The corresponding HS‐free systems, CA + Urea + BaCl_2_ and CA + Urea + ZnCl_2_, show slightly lower QY values of 60.1% and 48.2%, respectively. In contrast, the undoped HS‐containing samples, HS_CA+Urea_, CA + Urea, and HS_Urea_ exhibit QY values of 39.7%, 36.9%, and 31.7%, respectively.

This systematic comparison clearly demonstrates that while citric acid and urea can independently generate emissive CQDs, the incorporation of hazelnut shell and metal ions synergistically enhances the optical efficiency. The presence of hazelnut shell contributes to improved surface passivation and a more favorable distribution of emissive states, while metal‐ion assistance further promotes structural ordering and suppresses nonradiative recombination pathways.

Taken together, these results demonstrate that the HS_CA+Urea+BaCl2_ and HS_CA+Urea+ZnCl2_ formulations yield the most structurally uniform and optically active CQDs, characterized by improved graphitic domains, reduced surface defect density, and enhanced quantum yield compared with both undoped and HS‐free systems. Consequently, these two optimized systems were selected for all subsequent structural, morphological, and electro‐optical investigations presented in the following sections.

**FIGURE 4:**
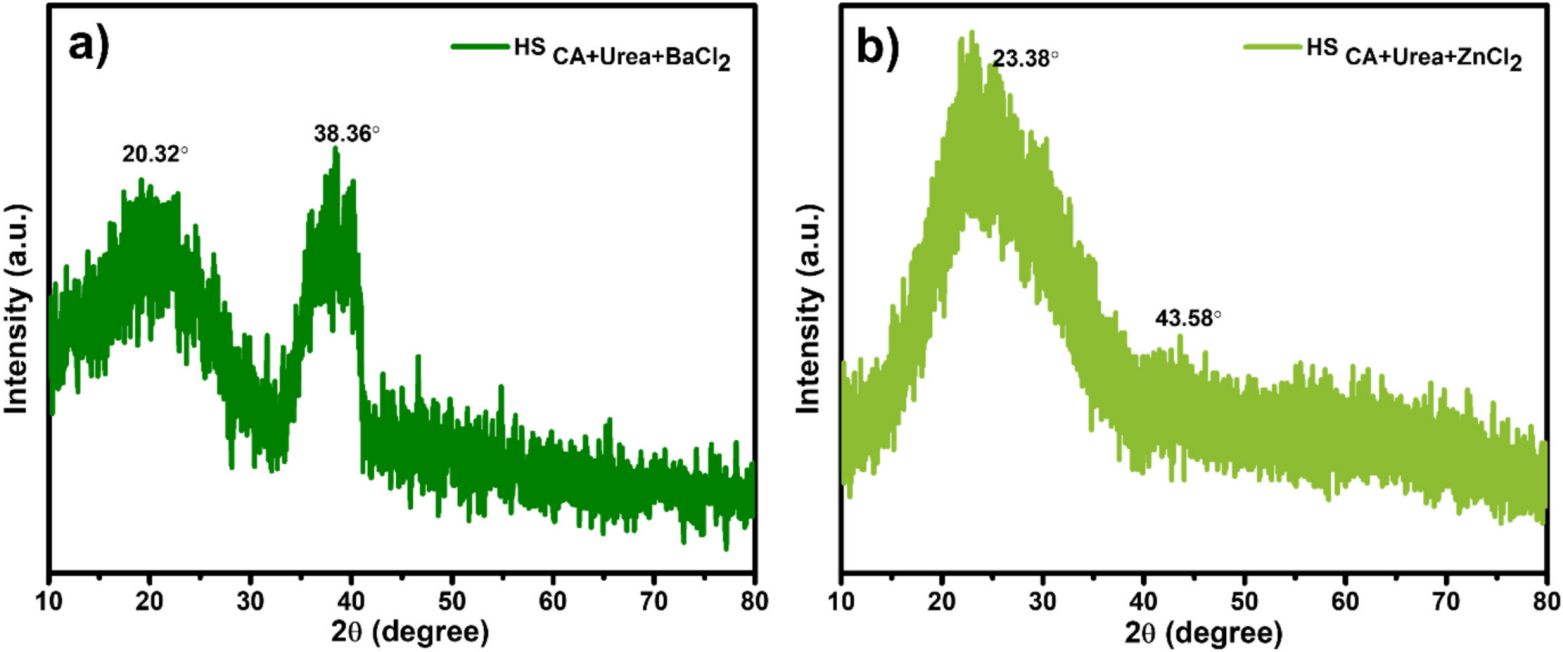
XRD patterns of (a) HS_CA+Urea+BaCl2_ and (b) HS_CA+Urea+ZnCl2_.

The crystalline structure of the HS_CA+Urea+BaCl2_ and HS_CA+Urea+ZnCl2_ derived CDs was investigated using powder XRD, as shown in Figure 4a,b. The BaCl_2_‐assisted sample (Figure 4a) exhibits two broad diffraction peaks centered at approximately 2*θ* = 20.3° and 38.4°, which can be assigned to the (002) and (100) planes of turbostratic graphitic carbon, respectively, in accordance with JCPDS No. 41‐1487. The lower angle (002) reflection indicates an expanded interlayer spacing typical of disordered graphitic domains, whereas the higher angle (100) peak corresponds to in‐plane ordering of sp^2^ carbon networks. The relatively sharp feature at 38.4° suggests that Ba^2+^ ions promote localized ordering and the development of short‐range graphitic domains, consistent with their catalytic role in enhancing carbonization and facilitating the rearrangement of sp^2^ carbon networks during thermal treatment [32, 33, 34]. In contrast, the ZnCl_2_‐derived sample (Figure 4b) exhibits a single, broad diffraction halo centered at 2*θ* ≈ 23.4°, characteristic of amorphous carbon structures with an expanded interlayer spacing (*d*_002_ ≈ 0.38 nm) compared with bulk graphite (0.34 nm). This broad feature reflects a low degree of structural ordering, consistent with a defect‐rich carbon matrix and the retention of oxygenated surface groups, as supported by FTIR analysis. A weak additional feature around 43.6° can be attributed to disordered sp^2^ domains or graphitic (100) reflections with significantly reduced crystallite size [35, 36]. Together, these findings indicate that BaCl_2_ promotes more ordered turbostratic graphitic domains, whereas ZnCl_2_ favors the formation of highly amorphous, heteroatom‐rich carbons. This structural disparity correlates with the higher PL QY and absorbance intensity of the BaCl_2_‐assisted sample, because graphitic ordering enhances electronic delocalization and radiative recombination efficiency.

**FIGURE 5:**
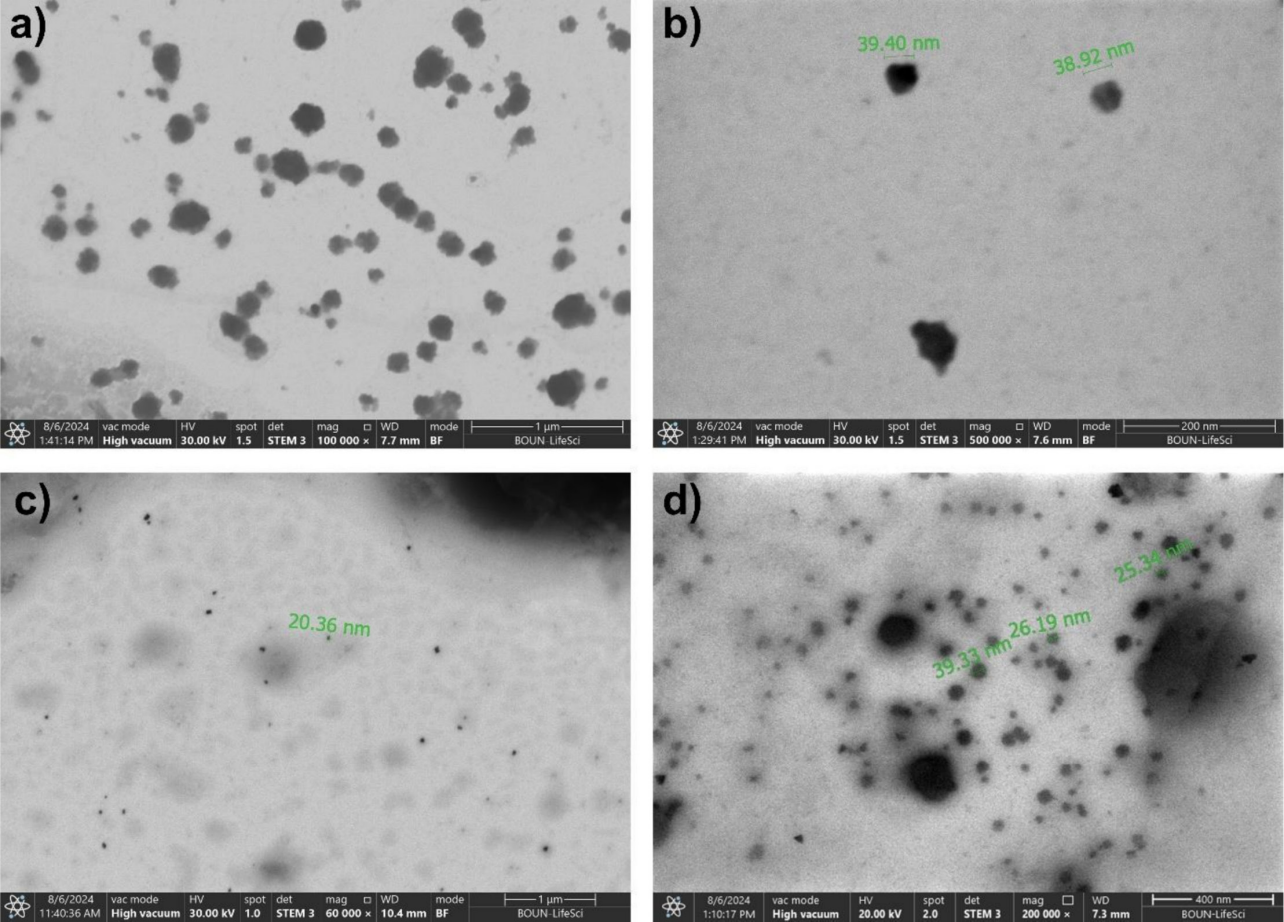
STEM images and corresponding DLS analyses of CQDs synthesized from lignocellulose: (a,b) STEM images of HS_CA+Urea+BaCl2_ and (c,d) STEM images of HS_CA+Urea+ZnCl2_.

The morphological characteristics of the CQDs synthesized using HS_CA+Urea+BaCl2_ and HS_CA+Urea+ZnCl2_ were investigated by STEM, as presented in Figure 5a–d. The BaCl_2_‐assisted sample (Figure 5a,b) exhibits well‐dispersed, nearly spherical nanoparticles with relatively uniform size distribution. High‐magnification images confirm an average particle size in the range of ~38–40 nm, consistent with the formation of larger, partially graphitized carbon domains, which is in line with the sharper (002) diffraction peak observed in the XRD pattern (Figure 4a). The particles appear with distinct contrast, suggesting denser cores and enhanced electron scattering, which may be attributed to the presence of Ba^2+^ residues or the higher degree of carbon ordering [37].

In contrast, the ZnCl_2_‐assisted sample (Figure 5c,d) reveals a broader size distribution with a higher fraction of ultrasmall nanoparticles (15–25 nm) interspersed with slightly larger aggregates (up to ~39 nm). The overall morphology appears more diffuse with lower contrast, indicative of a less crystalline, more amorphous carbon network. This observation is consistent with the broad halo in the XRD pattern (Figure 4b) and the stronger O–H/N–H and C–O stretching bands in FTIR, implying that ZnCl_2_ promotes the retention of heteroatom‐rich surface functionalities and prevents extensive graphitic stacking. The presence of multiple size populations suggests that ZnCl_2_ may act as a porogen, facilitating the generation of defect‐rich, highly emissive surface states that contribute to the observed blue‐shifted PL emission [38, 39].

Taken together, these STEM results highlight the distinct roles of BaCl_2_ and ZnCl_2_ during carbonization: While BaCl_2_ promotes growth and ordering of carbon nuclei, leading to slightly larger and more crystalline CQDs, ZnCl_2_ favors a more heterogeneous nucleation process that yields smaller, less ordered, and chemically diverse nanoparticles. Both types of CQDs exhibit blue PL, confirming the formation of carbonaceous domains with similar electronic structures; however, BaCl_2_‐derived CQDs display noticeably stronger emission intensity, consistent with a lower density of nonradiative defect states and better surface passivation. In contrast, ZnCl_2_‐derived CQDs, with their more disordered and heterogeneous surfaces, produce weaker emission but may offer tunable optical properties through surface‐state engineering.

**FIGURE 6:**
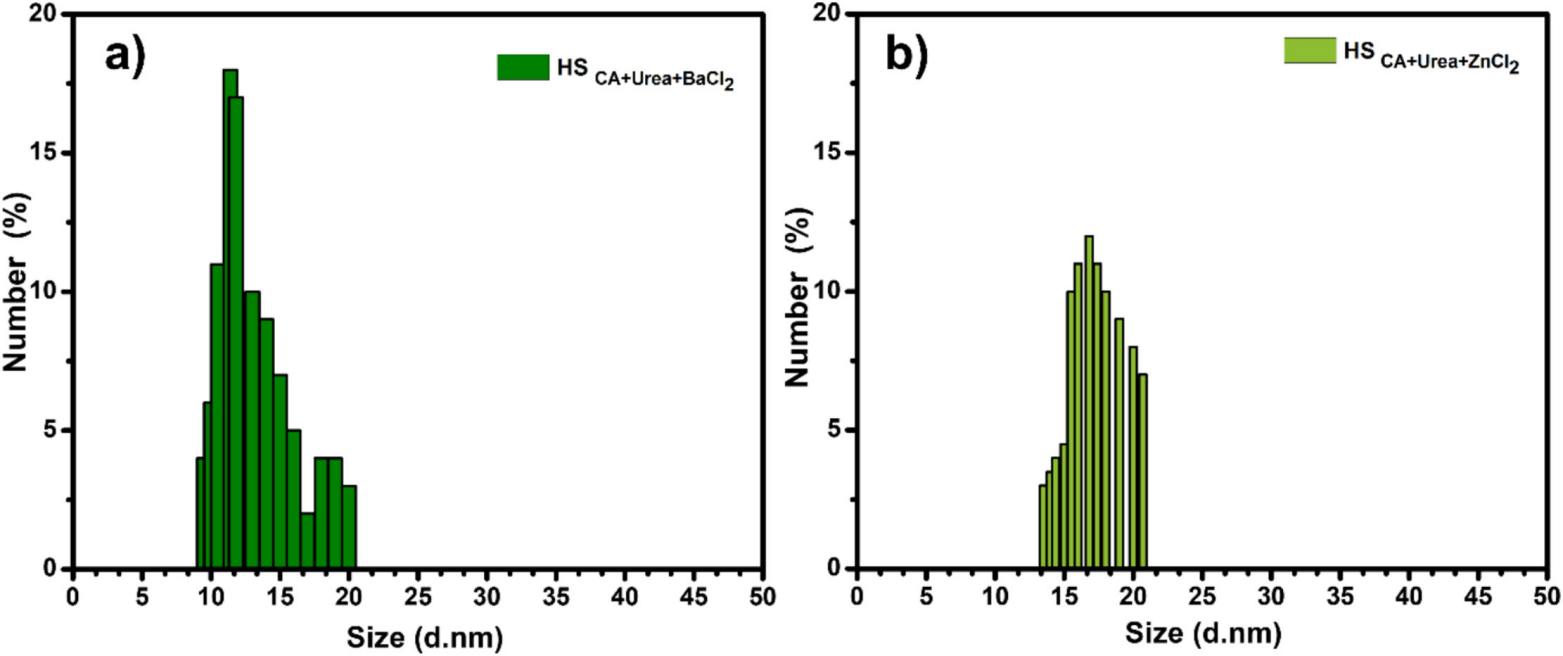
DLS size distribution graphs of HS_CA+Urea+BaCl2_ and of HS_CA+Urea+ZnCl2_, respectively.

The number‐based DLS histograms reveal narrow, essentially unimodal hydrodynamic size distributions for both samples, indicative of good colloidal uniformity and minimal aggregation in water. For HS_CA+Urea+BaCl2_ (Figure 6a), the main population spans ~10–21 nm with a clear mode around ~11–13 nm. HS_CA+Urea+ZnCl2_ (Figure 6b) is centered at slightly larger sizes, ~14–21 nm with a mode near ~16–19 nm. Because DLS reports the hydrodynamic diameter (core + solvation layer + any adsorbed ligands), these sizes represent the dispersed fraction in solution; they need not coincide with the larger features occasionally seen by STEM (e.g., sporadic 25‐ to 40‐nm objects), which likely include small aggregates or high‐contrast clusters. The narrow DLS peaks are consistent with the FTIR evidence for abundant O–H/N–H and C–O groups that provide electrostatic/steric stabilization, suppressing secondary aggregation. The modest right‐shift for the HS_CA+Urea+ZnCl2_ sample agrees with its more heteroatom‐rich surface (stronger C–O band) and higher surface solvation, which increases hydrodynamic radius, while the HS_CA+Urea+BaCl2_ sample's slightly smaller DLS mode but more ordered (turbostratic) cores (XRD) and higher PL QY suggest fewer surface traps and tighter carbon domains dispersed as smaller solvated units. Overall, DLS corroborates a well‐dispersed colloidal state for both CQDs, with ZnCl_2_ yielding a marginally larger hydrodynamic envelope and BaCl_2_ giving a comparably narrow but left‐shifted distribution.

**FIGURE 7:**
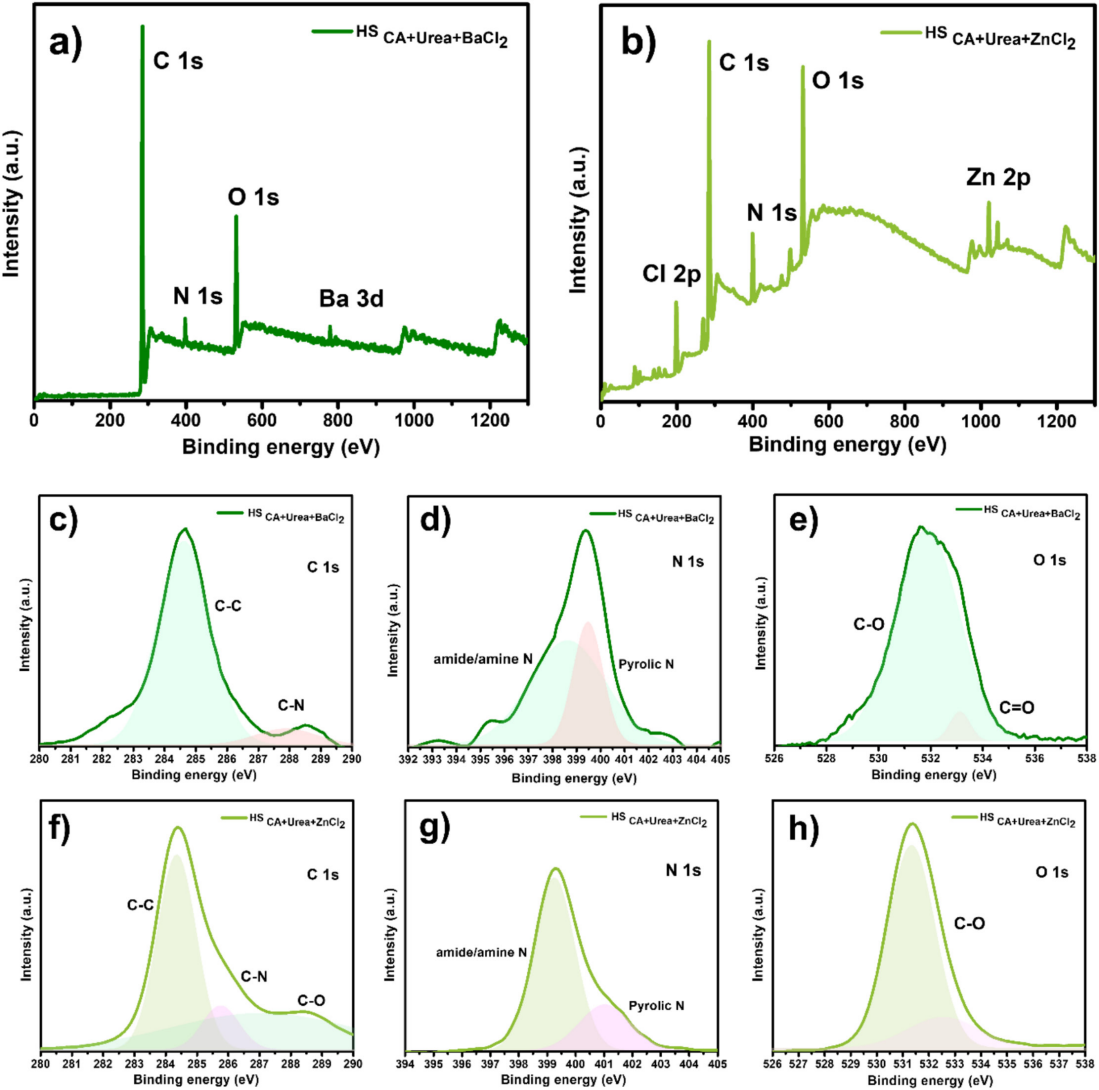
XPS analysis of CQDs synthesized with BaCl_2_ and ZnCl_2_. (a,b) Survey spectra showing C, N, O, and respective metal signals. (c,f) C 1s spectra, (d,g) N 1s spectra, and (e,h) O 1s spectra.

The surface elemental composition and chemical bonding states of BaCl_2_‐ and ZnCl_2_‐assisted CQDs were comprehensively analyzed by XPS (Figure 7). The survey spectra (Figure 7a,b) clearly show the presence of C 1s, N 1s, and O 1s peaks as the dominant components for both samples, confirming the carbonaceous nature of the CQDs and the successful incorporation of heteroatoms originating from citric acid and urea. In addition, Ba 3d and Zn 2p signals are observed in the BaCl_2_‐ and ZnCl_2_‐assisted CQDs, respectively, along with Cl 2p peaks, indicating the presence of residual metal chloride species. Given the relatively large ionic radii of Ba^2+^ and Zn^2+^ and the turbostratic/amorphous nature of the CQD network, these species are most likely surface‐bound or partially trapped at the interface, rather than substitutionally doped into the carbon lattice.

High‐resolution C 1s spectra (Figure 7c,f) are dominated by a main peak at approximately 284.6 eV, attributed to sp^2^‐hybridized C–C/C = C bonds, indicative of conjugated graphitic domains. Secondary components at ~285.7 and ~287.8 eV correspond to C–N/C–O and C = O/C–O–C species, respectively. The ZnCl_2_‐derived CQDs display a relatively stronger C = O contribution, consistent with a more oxygenated and defect‐rich surface chemistry compared with their BaCl_2_‐derived counterparts [40].

The N 1s spectra (Figure 7d,g) of both samples exhibit two main contributions: amide/amine nitrogen at ~399.4 eV and pyrrolic nitrogen at ~400.9 eV. Notably, the ZnCl_2_‐assisted CQDs show a slightly higher proportion of pyrrolic nitrogen, suggesting a greater density of defect‐related nitrogen species, which can act as emissive trap states and influence photophysical properties, such as inducing a slight red‐shift and broadening of PL emission [41, 42, 43].

The O 1s spectra (Figure 7e,h) further support these observations. BaCl_2_‐assisted CQDs exhibit a dominant C–O peak (~532.2 eV) and a relatively weak C = O component (~533.5 eV), indicative of a less oxidized surface and higher structural order. In contrast, the ZnCl_2_‐assisted CQDs display a broader O 1s envelope with a stronger C = O contribution, reflecting a more oxidized surface rich in defect sites. This chemical environment is consistent with the FTIR and XRD findings, which suggest enhanced graphitic ordering in the BaCl_2_‐derived samples and increased disorder in the ZnCl_2_‐derived ones [44, 45, 46].

Overall, the XPS results reveal a clear distinction between the two systems: BaCl_2_‐assisted CQDs possess a more graphitic and less defect‐prone surface structure, which correlates with their higher PL QY (63.4%) and superior OLED luminous efficiency (LE). In contrast, ZnCl_2_‐assisted CQDs exhibit a more oxygen‐ and nitrogen‐rich, defect‐dominated surface, which is associated with lower QY and reduced device efficiency despite their higher luminance. These findings underscore the critical role of metal ion–assisted nucleation in tailoring the surface chemistry and optoelectronic performance of CQDs.

To further clarify the role of metal ions during the carbonization process, it is important to note that Ba^2+^ and Zn^2+^ do not behave as classical lattice dopants but rather act as structure‐directing and surface‐modifying agents during thermochemical conversion. Due to their relatively large ionic radii and ionic nature, these species are primarily localized at the surface or interfacial regions of the forming carbon domains, as supported by XPS analysis.

Ba^2+^ ions promote carbonization and facilitate the rearrangement of sp^2^ carbon networks, acting as a graphitization‐enhancing agent that leads to more ordered turbostratic structures with reduced defect density. In contrast, Zn^2+^ ions tend to stabilize oxygen‐ and nitrogen‐containing functional groups, favoring the formation of a more amorphous, heteroatom‐rich carbon matrix. This behavior suggests that Zn^2+^ functions as a surface‐state regulator, increasing defect density and enabling a broader distribution of emissive sites.

Thus, the distinct optoelectronic properties observed for BaCl_2_‐ and ZnCl_2_‐assisted CQDs arise from their different roles during nucleation and carbonization: Ba^2+^ primarily acts as a structure‐directing catalyst promoting graphitic ordering, whereas Zn^2+^ serves as a surface‐modifying agent that enhances defect‐related emissive states.

**FIGURE 8:**
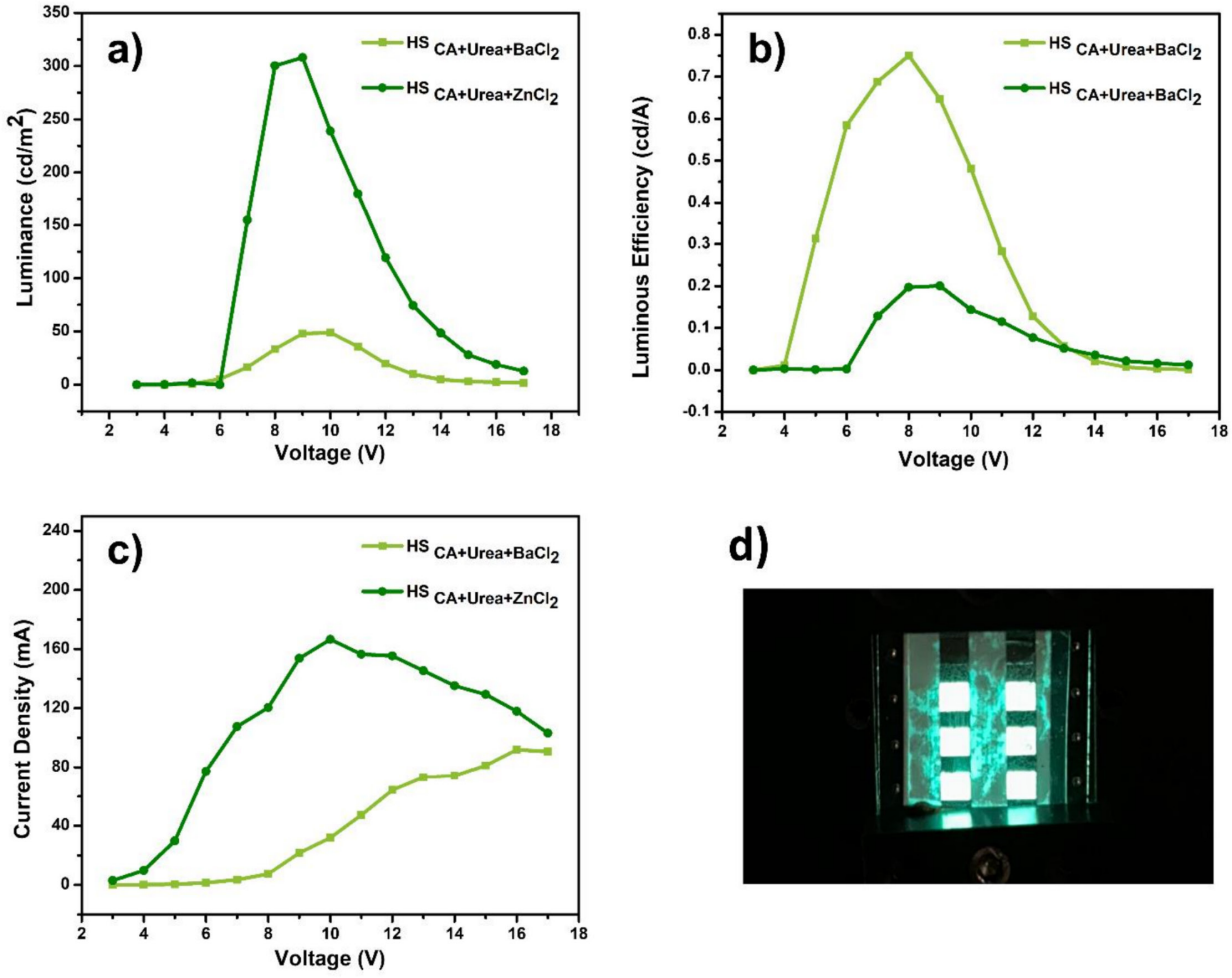
(a) Luminance–voltage, (b) luminous efficiency–voltage, and (c) current density–voltage characteristics of the devices fabricated with HS_CA+Urea+BaCl2_ and HS_CA+Urea+ZnCl2_. (d) The photo of the OLED device which contains HS_CA+Urea+ZnCl2_.

The OLED devices based on the synthesized CDs are fabricated and characterized, the graphs of the device characteristics are shown in Figure 8 and summarized in Table 1. The electroluminescent performance of the HS_CA+Urea+BaCl2_‐based OLEDs exhibited a strong dependence on the incorporated metal chloride groups. As shown in Table 1, the HS_CA+Urea+BaCl2_ device demonstrated a lower turn‐on voltage (5 V) compared with the HS_CA+Urea+ZnCl2_ counterpart (7 V), indicating more efficient charge injection and transport within the emissive layer. This improvement can be attributed to the ionic radius and electronic configuration of Ba^2+^, which may facilitate better energy level alignment and enhance the formation of interfacial dipoles at the emissive‐transport layer interface.

**TABLE 1:** Summary of the OLED performances of the devices fabricated based on HS_CA+Urea+BaCl2_ and HS_CA+Urea+ZnCl2_.

Sample	Turn‐on voltage (V)	EQE (%)	CIE coordinates (*x*, *y*)	L (cd m^−2^)	LE (cd A^−1^)
HS_CA+Urea+BaCl2_	5	0.22	(0.32, 0.53)	48.9	0.75
HS_CA+Urea+ZnCl2_	7	0.064	(0.32, 0.53)	308.1	0.20

Although the HS_CA+Urea+BaCl2_ device displayed a relatively lower maximum luminance (48.9 cd m^−2^) compared with the ZnCl_2_‐based device (308.1 cd m^−2^), it achieved significantly higher EQE (0.22%) and LE (0.75 cd A^−1^). This suggests that the BaCl_2_ incorporation improves radiative recombination probability by reducing nonradiative pathways, possibly through improved exciton confinement or defect passivation. Conversely, the HS_CA+Urea+ZnCl2_ device, despite its higher luminance output, suffered from lower EQE (0.064%) and LE (0.20 cd A^−1^), which may arise from enhanced leakage current or imbalanced carrier transport leading to exciton quenching.

Both devices exhibited identical CIE coordinates of (0.32, 0.53), corresponding to green emission. The stability of the chromaticity regardless of dopant composition suggests that the emission originates predominantly from the CD emissive centers rather than from metal‐related transitions. Overall, these results highlight the crucial influence of the metal chloride dopant on charge balance and recombination dynamics, demonstrating that BaCl_2_ doping yields more efficient but lower intensity emission, whereas ZnCl_2_ incorporation favors higher brightness at the expense of quantum efficiency.

## 5 Conclusion

This work reports a sustainable solid‐state synthesis route for N‐CQDs derived from hazelnut shell, using BaCl_2_ and ZnCl_2_ doping to modulate nucleation, crystallinity, and surface chemistry. Structural analyses confirmed that BaCl_2_‐assisted CQDs were smaller (7–13 nm), highly graphitized, and exhibited well‐defined morphology, whereas ZnCl_2_‐assisted CQDs were larger (≈20–23 nm), more amorphous, and oxygen rich. XPS data revealed that BaCl_2_‐doping resulted in surfaces dominated by graphitic C–C/C = C and C–N bonds, while ZnCl_2_ induced the formation of C–O/C = O species, increasing surface polarity and defect density. These structural differences translated into distinct optical characteristics: BaCl_2_‐CQDs displayed sharp π–π* absorption at ~280 nm, strong blue emission at 405 nm, and a quantum yield of 63.4%, compared with the slightly red‐shifted emission (412 nm) and lower QY (50.1%) of ZnCl_2_‐CQDs. Both systems exhibited excellent photostability, retaining > 95% of PL intensity after 30 days. When integrated as emissive layers in OLED devices, BaCl_2_‐CQDs delivered superior performance, achieving a luminance of 48.9 cd m^−2^ and a current efficiency of 0.75 cd A^−1^, outperforming ZnCl_2_‐CQDs (308 cd m^−2^ and 0.20 cd A^−1^). This performance is attributed to improved crystallinity, reduced trap‐state density, and more efficient charge recombination in Ba‐doped CQDs. Overall, this study highlights metal ion–assisted nucleation as a powerful design strategy to tailor CQD structure and optoelectronic properties, offering a green and scalable pathway toward high‐efficiency, durable, and environmentally responsible OLED technologies.

## Author Contributions

**Fatmanur Uyumaz Cengiz:** investigation, methodology, writing – original draft, visualization. **Figen Türksoy:** methodology, writing – original draft, resources. **Emine Tekin:** methodology, writing – original draft, resources. **Güldem Utkan:** writing – review and editing, methodology. **Erhan Şükrü Cengiz:** methodology, writing – original draft, investigation. **Görkem Yumuşak:** writing – review and editing, methodology. **Memet Vezir Kahraman:** writing – review and editing, project administration, resources, supervision, methodology, conceptualization.

## Funding

This work was supported by the Scientific and Technological Research Council of Türkiye (TÜBİTAK) under the 1004—Centre of Excellence Support Program (Project No: 22AG043).

## Conflicts of Interest

The authors declare no conflicts of interest.

## Data Availability

Data sharing is not applicable to this article as no datasets were generated or analyzed during the current study.
